# ScRNAseq Analysis of Chicken Embryonic Pituitary Reveals Cell Heterogeneity and a Cell Type Coexpressing *Gh* and *Pomc*

**DOI:** 10.1210/endocr/bqaf184

**Published:** 2025-12-18

**Authors:** Kuan Ling Liu, Tom E Porter

**Affiliations:** Department of Animal and Avian Sciences, University of Maryland, College Park, MD 20742, USA; Animal Biosciences and Biotechnology Laboratory, Beltsville Agricultural Research Center, Agricultural Research Service, US Department of Agriculture, Beltsville, MD 20706, USA; Department of Animal and Avian Sciences, University of Maryland, College Park, MD 20742, USA

**Keywords:** pituitary, somatotrophs, corticotrophs, cortico-somatotrophs, growth hormone, proopiomelanocortin

## Abstract

Gene expression profiles and the heterogeneity among hormone-producing pituitary cells remain poorly characterized in most vertebrates, especially in chicken embryos. Using single-cell RNA sequencing, the transcriptomes of 4346 basal and 10 835 corticosterone (CORT)-treated embryonic day 11 chicken pituitary cells were sequenced. Classical endocrine cell clusters were identified, and some were shown to express previously unreported marker genes. A cluster of uncommitted cells was identified that expressed markers for multiple endocrine cell types, with ∼30% coexpressing *Gh* and *Pomc* mRNA. We named this population of cells the cortico-somatotrophs. The existence of cortico-somatotrophs were confirmed at both the mRNA and protein level. We further characterized the corticosomatotrophs by utilizing the known effect of CORT to increase somatotroph abundance. Identification of cortico-somatotrophs challenges the prevailing view that corticotrophs and somatotrophs develop from distinct cell lineages.

The pituitary gland serves as the central hub of the endocrine system, responsible for regulating various physiological processes including stress responses, growth, reproduction, and metabolism (1, 2). It has been well established that the anterior pituitary (AP), also known as the adenohypophysis, contains multiple distinct hormone-secreting cell populations. Each cell type is believed to secrete 1 or 2 hormones that are vital to these physiological processes (3, 4). The 5 primary cell types in the AP include the following: corticotrophs, which secrete ACTH; somatotrophs, responsible for producing GH; lactotrophs, which release prolactin; thyrotrophs, which produce TSH; and gonadotrophs, which produce LH and FSH (2, 5). In addition to the endocrine cell population, several nonendocrine cell types have also been discovered in the AP. These include proliferating cells, endothelial cells, and folliculo-stellate cells, which contribute to the overall function and structure of the gland (6, 7).

While the cellular composition and function of the pituitary gland have been well studied in vertebrates, the gene expression profiles of individual pituitary cell populations remain poorly characterized. Advancements in single-cell RNA sequencing (scRNAseq) have provided transcriptomic data for specific pituitary cell types in several vertebrates, including humans (8, 9), rats (10), mice (11-13), chickens (14), zebrafish (15), and medaka (16). Although pituitary scRNAseq studies have been conducted in embryos of human (8) and mouse (17), comparable analyses in the chicken are lacking. In this study, we utilized scRNAseq to map the transcriptional profiles of chicken embryonic pituitary (CEP) cells. Our findings provide insights on differentially expressed genes (DEGs), heterogeneity of the endocrine cells, and a new pituitary cell type, the cortico-somatotrophs.

## Materials and Methods

### Cell Dissociation and Library Preparation

Fertilized Ross 308 chicken eggs used for the experiments were obtained from Perdue Hatchery (Hurlock, MD). The eggs were set in a humidified incubator (60% humidity, 37.5 °C), with day 1 of incubation denoted as e0. Pituitaries were dissected from e11 embryos, trypsin-dispersed, and pooled as previously described (18). Cell viability was assessed by the trypan blue dye-exclusion method (19). An individual e11 pituitary gland yielded around 300 000 cells, and cell viability was >95% in all experiments. Dispersed cells were plated at a density of 1 000 000 cells per well in poly-L-lysine-coated 24-well plates for 1 hour, then supplemented with DMEM/F12 medium with 0.1% BSA, 100 U/mL penicillin, and 100 μg/mL streptomycin for 19 hours. Corticosterone (CORT; 100 nM final concentration) was added to the appropriate wells for an additional 6 hours. The cells were collected using 0.25% trypsin-EDTA (ThermoFisher, Waltham, MA), washed twice with chilled Dulbecco's phosphate-buffered saline, and resuspended in chilled Dulbecco's phosphate-buffered saline (1 × 10^6^ cells per 200 μL). The cells were methanol-fixed based on the protocol provided by Genewiz from Azenta Life Sciences and then further processed by their Next-Generation Sequencing team. They estimated the cell viability and numbers, produced the cDNA libraries, and sequenced the filtered cells on the Illumina Series platform (Genewiz, Summerfield, NJ).

### Sequencing Data Processing and Clustering

For RNA sequencing analysis of single pituitary cells, Azenta Life Sciences generated the quality control reports (Fig. S1) (20), the FASTQ raw data files (21), and the interactive differential gene expression analysis with Loupe Browser v8.0.0 (10× Genomics, Pleasanton, CA) (21). The quality reports reveal information such as cell quality, sequencing depth, and genome alignment (Fig. S1) (20). Azenta further converted both FASTQ files to genecount matrix with the cell barcodes aligned in reference to the GRCg6a transcriptome from the Ensembl database: Ensembl release 105. The filtered counts matrix and associated metadata processed through Cell Ranger v7.0 were imported to Seurat package v4.2.0 in R to clusters and further analyzed, using default parameters unless specified otherwise (22-26).

The difference in cell number between basal (4525 cells) and CORT-treated (11 842 cells) conditions reflects technical variation in sequencing performance. To compensate for a lack of sequencing depth in the CORT-treated samples, a greater number of CORT-treated cells were captured during sequencing to ensure comparable overall sequencing coverage across conditions. We minimized the impact of this difference in cell numbers by performing downstream analysis based on the percentage of cells rather than relying solely on cell counts.

We first filtered out cells with lower than 500 counts of unique molecular identifier or lower than 200 unique genes detected. We then removed cells containing more than 5% mitochondrial RNA reads or 25% ribosomal RNA reads. Doublets that may affect clustering analysis were detected and removed using the R package DoubletFinder v.2.0.3 with default command line options; assuming 3.5% and 9% doublet formation rate for basal and CORT-treated cells, respectively (27). Post-quality control, 2746 basal (60.69%) and 8843 CORT-treated (74.67%) cells were used for further analysis. The remaining data were normalized based on standard workflow and variable features were identified. Data dimensionality reduction was performed using principal component analysis. Using the “FindCluster” function, with 15 principal components and a resolution of 0.8, the cells were partitioned into distinct clusters. Clustered datasets were converted to a csv file and further analyzed using Loupe Browser v8.0.0. Using a combination of known marker genes identified from the literature, cell types were determined based on the corresponding log_2_exp threshold (Table 1). Each log_2_exp threshold was adjusted by determining the lowest marker gene expression for each target cell cluster that excludes background expression (Figs. S2 and S3) (20). Clusters that appeared to belong to the same cell type were further combined and then visualized using the Uniform Manifold Approximation and Projection (UMAP; Fig. S4) (20, 28).

**Table 1.** Percent population of the identified 6 hormone-producing cell clusters from day 11 embryonic chicken pituitary under basal and CORT-treated conditions

Cell clusters	Number of cells		Cell population (%)	
	Basal	CORT	Basal	CORT
Corticotrophs	113	445	7.87	7.80
Gonadotrophs	200	1073	13.94	18.80
Thyrotrophs	171	531	11.92	9.30
Somatotrophs	566	2618	39.44	45.87
Mammosomatotrophs	20	118	1.39	2.07
Premature nebulous	365	922	25.44	16.16
Total	1435	5707	100.00	100.00

Abbreviation: CORT, corticosterone.

### DEGs and Analysis

DEGs were identified by the “Run Differential Expression” function in Loupe Browser. Features expressed highly within each cluster, relative to the entire dataset, were identified. Significant DEGs were selected from upregulated genes with adjusted *P*-value <.05 and an average occurrence of greater than 1 count per cell across the entire dataset. The complete lists of DEGs are included in Datasets S1 and S2 (29).

### Merging Datasets

Subsets of basal and CORT datasets were merged and reanalyzed to determine the CORT effect on different cell types. The basal and CORT clusters for each hormone-producing cell type were combined and then normalized based on standard workflow to check for batch effects. Data dimensionality reduction was performed using principal component analysis. Using the “FindCluster” function, with 7 principal components and a resolution of 0.8, the cells were partitioned into distinct clusters. The clusters were reassigned back into either basal or CORT-treated groups, and then integration was performed to correct for batch effects. The datasets were visualized as UMAPs, and DEGs from treatment effect were determined using the “FindMarker” command. Features with an adjusted *P-*value <.05 were determined.

### RNA Fluorescence In Situ Hybridization

Following the HCR™ RNA fluorescence in situ hybridization (FISH; Molecular Instruments, Los Angeles, CA) company protocol (30, 31), mRNA expression of selected genes was characterized in different hormone-producing cell types. Pituitaries were collected from e11 chicken embryos (n = 60), divided evenly into 3 separate tubes, and then trypsin-dispersed into cells. Each of the individual cell replicates (n = 3) were separately plated at a density of 3000 cells per well on poly-L-lysine coated 12 mm diameter circular glass coverslips (FisherScientific, Hampton, NH) and allowed to attach for 3 hours at 37.5 °C. DMEM/F12 medium supplemented with 0.1% BSA, 100 U/mL penicillin, and 100 μg/mL streptomycin was added, and the cells were cultured for an additional 19 hours. Cells were treated with or without CORT (100 nM final concentration) for an additional 6 hours and then fixed in 4% formaldehyde in PBS for 20 minutes at room temperature (RT). The cells were left in formaldehyde at 4 °C overnight then washed 3 times with PBS at RT. After the last wash, the cells were permeabilized with ice-cold 70% ethanol overnight at -20 °C. Cells were washed with 2× sodium chloride sodium citrate (SSC) and pre-hybridized with the probe hybridization buffer for 30 minutes at 37 °C. The probe hybridization buffer was replaced with the probe solution (4 nM), and the cells were incubated overnight at 37 °C. Custom probes were made for chicken *Pomc* (NM_001398117.1; B2 amplifier) and *Gh* (NM_204359.2; B3 amplifier) mRNA, with 20 sets of probes for each gene. Excess probes were removed using the probe wash buffer at 37 °C, and the cells were further washed using 5× sodium chloride sodium citrate with 0.1% Tween-20. To amplify the signal, cells were preamplified in the amplification buffer for 30 minutes at RT and then incubated in the hairpin mixture (60 nM) overnight (>12 hours) in the dark at RT. We used the B2 amplifier with 546 nm fluorophore and the B3 amplifier with 488 nm fluorophore. The cells were further washed using 5× sodium chloride sodium citrate with 0.1% Tween-20 to remove excess hairpins, and the coverslips were reverse mounted back onto microscope slides using the DAPI Fluoromount-G® (SouthernBiotech, Birmingham, AL).

### Dual Label Immunofluorescence

Pituitaries were collected from day 11 chicken embryos (n = 70), divided evenly into 4 separate tubes, and then trypsin-dispersed into cells. Each of the individual cell replicates (n = 4) were separately plated at a density of 3000 cells per well on poly-L-lysine coated 12 mm diameter circular glass coverslips (FisherScientific, Hampton, NH) and allowed to attach for 3 hours at 37.5 °C. DMEM/F12 medium (ThermoFisher, Waltham, MA) supplemented with 0.1% BSA, 100 U/mL penicillin, and 100 μg/mL streptomycin was added, and the cells were treated with or without CORT (100 nM final concentration) and cultured for an additional 19 hours. After the cells were fixed in 1% formaldehyde for 20 minutes at RT, the slides were pretreated using 0.1% Tween-20/0.1% TX-100 followed by 0.3% H_2_O_2_. The cells were blocked using 2% normal donkey serum and then incubated in primary antibodies including the rabbit anti-GH (18) (1:1000) and mAb anti-ACTH (32) (1:1000) at 4 °C overnight. The cells were then incubated with secondary antibodies including Rhodamine (TRITC) AffiniPure™ Donkey Anti-Rabbit IgG (H + L) (Jackson ImmunoResearch Labs, West Grove, PA; catalog #: 711-025-152; RRID: AB_2340588; 7.5 μg/mL) and Fluorescein AffiniPure Donkey Anti-Mouse IgG (H + L) (Jackson ImmunoResearch Labs, West Grove, PA; catalog #: 715-095-150; RRID: AB_2340792; 7.5 μg/mL) at 4 °C overnight. Multiple PBS washes were done between treatments and antibody incubations. After the last PBS wash, coverslips were reverse-mounted back onto microscope slides using the DAPI Fluoromount-G® (SouthernBiotech, Birmingham, AL).

### Microscope Imaging and Processing

Microscope slides were imaged and further processed using a Nikon ECLIPSE Ti2 Inverted Microscope (Nikon, Tokyo, Japan). For RNA-FISH processed slides, under the 20× DIC objective lens, *Pomc*+ cells were visualized using the red channel (excitation: 555 nm; emission: 595 nm) an exposure time of 800 ms, *Gh*+ cells were visualized using the green channel (excitation: 475 nm; emission: 515 nm) with an exposure time of 1 seconds, and 4′,6-diamidino-2-phenylindole-positive (DAPI+) cells were visualized using the blue channel (excitation: 375 nm; emission: 432 nm) with an exposure time of 50 ms. The lookup tables thresholds were set from 5000 to 65 000. Images at 20× were selectively taken only if the visualized area contained 1 or more cells that were *Pomc*+/*Gh*+/DAPI+. The images were further deconvoluted to improve the estimate of the image intensity.

For dual label immunofluorescence processed slides, under the 20× DIC objective lens, ACTH+ cells were visualized using the red channel (excitation: 555 nm; emission: 595 nm) an exposure time of 2 seconds, GH+ cells were visualized using the green channel (excitation: 475 nm; emission: 515 nm) with an exposure time of 400 ms, and DAPI+ cells were visualized using the blue channel (excitation: 375 nm; emission: 432 nm) with an exposure time of 50 ms. The lookup tables thresholds were set from 6100 to 40 000. Images were selectively taken only if the visualized area contained 1 or more cells that were ACTH+/GH+/DAPI+. The brightness of the images was increased using Adobe Photoshop 2024 (Adobe, San Jose, CA).

### Quantification and Statistical Analysis

Three separate microscope images were captured for each treatment per biological replicate. A total of more than 200 cells (DAPI+) were counted to quantify the percent cell population for each cell type. Each experiment was replicated 4 times unless otherwise stated. The JMP statistical analysis system (JMP Statistical Discovery LLC, Cary, NC) was used to determine statistically significant differences among treatments or groups with a mixed-model ANOVA, where the replicate experiment was a random effect in the model. A post hoc test of least significant differences with Tukey's method for multiple comparisons was used to determine significant differences among groups. Different letters are used to denote significance at *P* < .05.

## Results

### ScRNAseq Revealed 12 Clusters in the AP

To investigate the transcriptome of CEP cell populations, embryonic day (e)11 AP glands from chicken embryos were dispersed, treated with or without CORT and analyzed through scRNAseq using the 10× Genomics system (33). This age (e11) and CORT treatment were chosen because we have previously reported that CORT can induce premature expression of *Gh* mRNA in e11 pituitary cells (34-42), and we were interested in identifying the somatotroph precursor population. A total of 4525 basal and 11 842 CORT-treated pituitary cells passed the initial quality control check; an average of 1333 genes and 33 188 transcripts were detected in basal cells, and an average of 855 genes and 13 366 transcripts were detected in CORT-treated cells (Fig. S1A and S1B) (20). These cells underwent additional filters, where cells expressing high levels of ribosomal and mitochondrial genes were further removed, as well as the doublets detected by DoubletFinder (Fig. S1C and S1D) (20). The remaining 2742 basal and 8843 CORT-treated cells were analyzed using Seurat to identify cell clusters, and uniform manifold approximation and projections (UMAPs) were generated for visualization.

**Figure 1.**
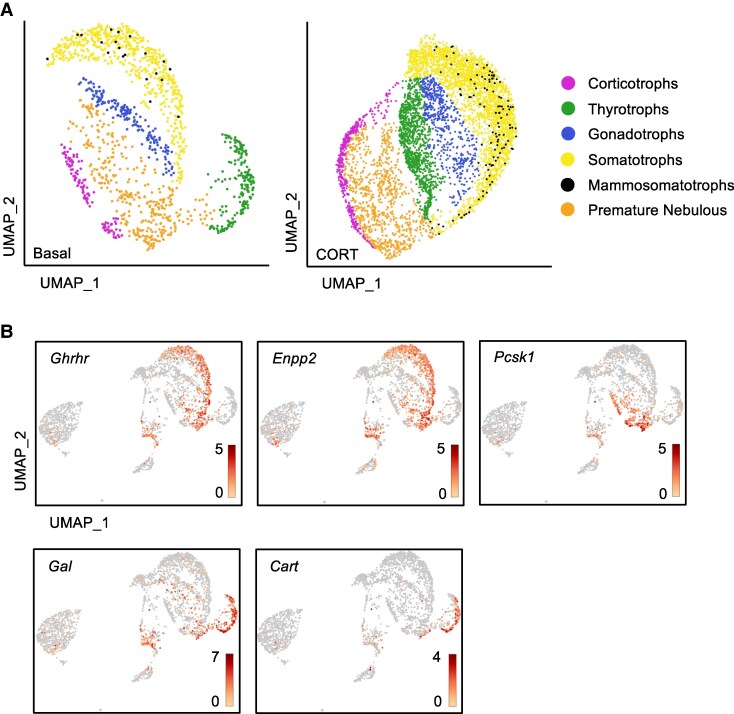
Uniform manifold approximation and projection visualization. (A) Six identified hormone-producing embryonic pituitary cell types based on the transcriptomes of 1437 basal cells and 5707 corticosterone-treated cells. Cells are colored by Loupe clustering and annotated by cell types (each point represents a single cell). (B) Expression level and distribution pattern of potential cell markers for somatotrophs (*Ghrhr* and *Enpp2*), corticotrophs (*Pcsk1*), and gonadotrophs (*Gal* and *Cart*). Expression levels are shown through the intensity of the red color scale. Cells in gray indicate no detection of the selected gene. Abbreviations: *Cart*, cocaine- and amphetamine-regulated transcript; *Enpp2*, ectonucleotide pyrophosphate/phosphodiesterase 2; *Gal*, galanin; *Ghrhr*, growth hormone-releasing hormone receptor; *Pcsk1*, proprotein convertase subtilisin/kexin type 1.

There were a total of 12 clusters, with 9 clusters identified using known marker genes (Fig. S4) (20). To better define cluster boundaries, the minimum expression level for each marker gene was individually selected (Figs. S2 and S3 and Table S1) (20, 43). Four classical hormone-producing cell types were identified, including somatotrophs, corticotrophs, gonadotrophs, and thyrotrophs (Fig. 1A). These cells were expressing *Gh*, *Pomc*, *Fsh* β subunit (*Fshb*), and *Tsh* β subunit (*Tshb*), respectively. There was not a clear lactotroph population; however, *Prl* expression was detected in some somatotrophs, so they are labeled as mammosomatotrophs (*Gh* and *Prl*). The expression levels and distribution of *Gh*, *Pomc*, *Fshb*, *Tshb*, and *Prl* were visualized through UMAPs (Fig. S5) (20). An additional hormone-producing cluster was identified to be expressing 2 or more different endocrine hormones, and it was named the premature nebulous (Fig. 1A). Nonendocrine or nonsecretory cells were also identified, including endothelial cells using TIMP metallopeptidase inhibitor 3 (*TIMP3*), folliculo-stellate cells using aldehyde dehydrogenase 6 (*ALDH6*), red blood cells using hemoglobin subunit alpha-A (*HBAA*), and white blood cells using the gene *ENSGALG00000029601* as marker genes (Fig. S4) (20). The endocrine/secretory cell clusters were isolated and further analyzed.

### Characterizing Hormone-producing Cells

Cell populations were determined for each hormone-producing cell cluster (Table 1). The largest basal cell population was the somatotrophs (39.44%), followed by premature nebulous (25.44%), gonadotrophs (13.94%), thyrotrophs (11.92%), corticotrophs (7.87%), and then mammosomatotrophs (1.39%). Most of the cell populations remained relatively similar under CORT treatment, with the exception of somatotrophs, gonadotrophs, and premature nebulous (Table 1). Somatotrophs and gonadotrophs increased by ∼5% while premature nebulous decreased by ∼9% of all pituitary cells. CORT treatment did not affect the nonendocrine cell populations (Table S2) (43). DEGs were identified in all clusters under both basal and CORT-treated conditions (Dataset S1 and S2) (29). Among the hormone-producing cell types, a few DEGs were identified to colocalize with their corresponding known marker genes, including growth hormone releasing hormone receptor (*Ghrhr*) and ectonucleotide pyrophosphate/phosphodiesterase 2 (*Enpp2*) in somatotrophs, proprotein convertase subtilisin/kexin type 1 (*Pcsk1*) in corticotrophs, and galanin (*Gal*) and cocaine- and amphetamine-regulated transcript (*Cart*) in gonadotrophs (Fig. 1B).

To determine the effect of CORT on hormone-producing cell clusters, each cell type from both basal and CORT-treated conditions was extracted and then merged together and reanalyzed for DEGs between treatment groups. Merged results were also visualized through UMAPs (Fig. S6) (20). Two DEGs were identified in both corticotrophs and somatotrophs (Table 2). In response to CORT, calcium voltage-gated channel auxiliary subunit alpha2delta (*Cacna2d1*) and heterogeneous nuclear ribonucleoprotein R (*Hnrnpr*) were upregulated corticotrophs, and neuropeptide Y (*Npy*) and neuron vesicle trafficking associated 1 (*Nsg1*) were upregulated in somatotrophs. No DEGs were identified in gonadotrophs, thyrotrophs, mammosomatotrophs, and cortico-somatotrophs (cells within the premature nebulous coexpressing *Gh* and *Pomc*).

**Table 2.** Differentially expressed genes of different hormone-producing cell types in response to corticosterone treatment on day 11 chicken embryonic pituitary cells

Cell type	Gene	*P*-value	log_2_FC	pct.1	pct.2	adj.*P*
Corticotrophs	*Cacna2d1*	8.8E-07	−0.32	0.190	0.704	.0018
Corticotrophs	*Hnrnpr*	5.6E-06	−0.26	0.458	0.965	.011
Somatotrophs	*Npy*	1.2E-07	0.32	0.604	1.000	.00023
Somatotroph	*Nsg1*	8.5E-07	−0.25	0.576	0.935	.0017

pct.1 is the proportion of cells that express the gene under basal conditions, and pct.2 is the proportion of cells that express the gene under corticosterone-treated conditions. No differentially expressed genes were identified for gonadotrophs, thyrotrophs, mammosomatotrophs, and cortico-somatotrophs. Adj.*P* or q < .05.

Abbreviations: adj.*P*, adjusted *P*-value; *Cacna2d1*, calcium voltage-gated channel auxiliary subunit α2delta; *Hnrnpr*, heterogeneous nuclear ribonucleoprotein R; *Npy*, neuropeptide Y; *Nsg1*, neuron vesicle trafficking associated 1.

### Heterogeneity among Pituitary Somatotrophs

**Figure 2.**
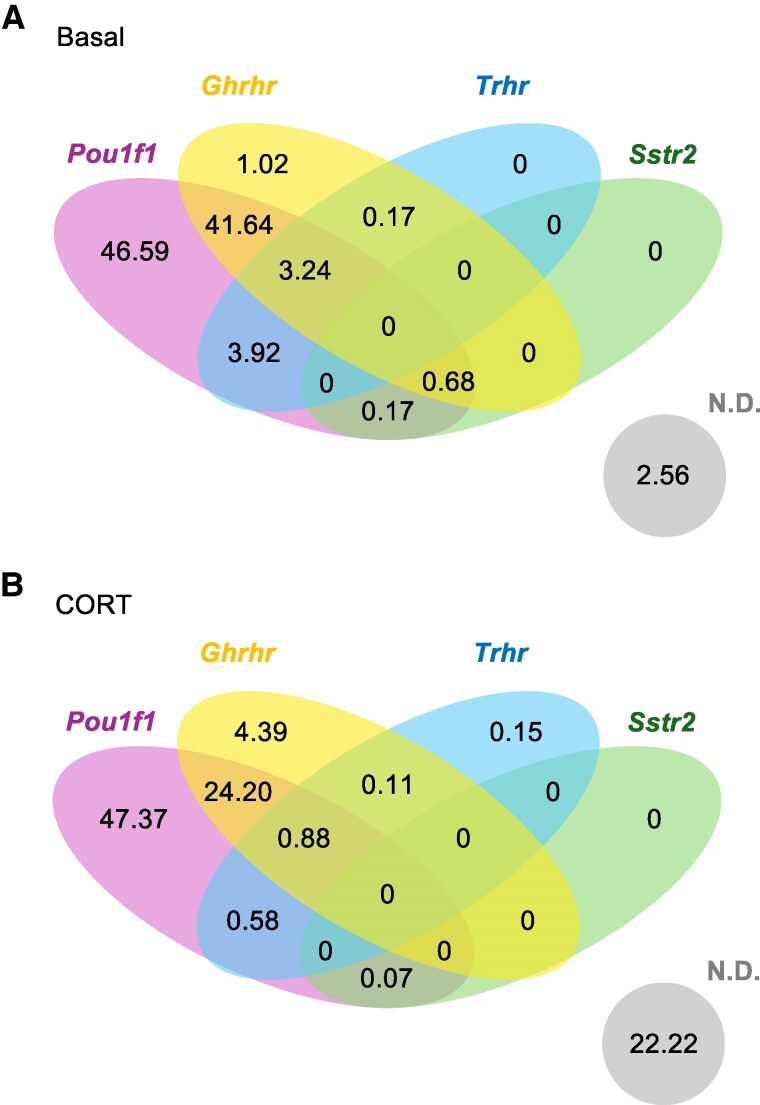
Venn diagram showing percentages of *Pou1f1*, *Ghrhr*, *Trhr,* and *Sstr2* expressing cells (≥log_2_1) in the somatotroph cluster for basal (A) and corticosterone-treated (B) day 11 chicken embryonic pituitary cells. Abbreviations: *Ghrhr*, growth hormone-releasing hormone receptor; N.D., not detected; *Pou1f1*, pituitary-specific transcription factor 1; *Sstr2*, somatostatin receptor 2; *Trhr*, thyrotropin-releasing hormone receptor.

**Figure 3.**
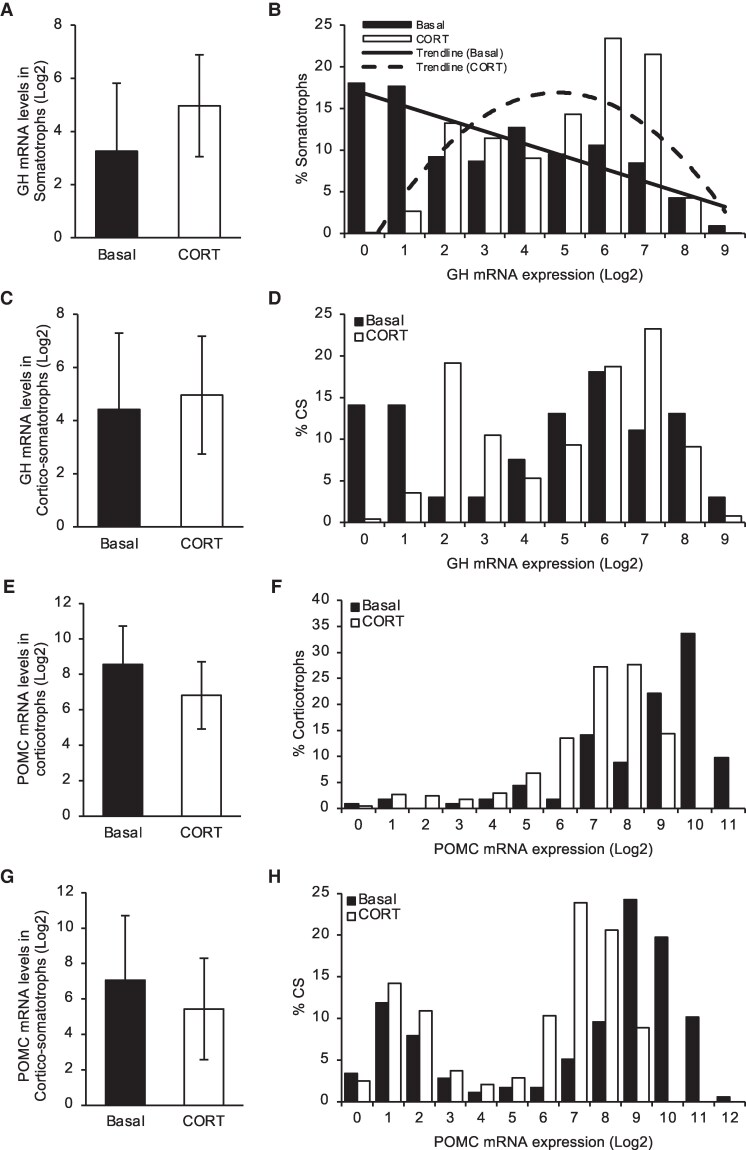
Somatotrophs, corticotrophs, and cortico-somatotrophs from embryonic day 11 chicken pituitary cells exhibited changes in mRNA levels following CORT treatment. (A and B) Somatotrophs revealed a shift in levels of *Gh* mRNA levels. (A) Average *Gh* levels shown in means +/− SE. (B) Percent of somatotrophs expressing different levels of *Gh*. A linear trendline (R^2^ = 0.7458) best fit the basal dataset, and a polynomial trendline (R^2^ = 0.6145) best fit the CORT-treated dataset. (E and F). Corticotrophs revealed a shift in *Pomc* mRNA. (E) Average *Pomc* levels shown in means +/− SE. (F) Percent of corticotrophs expressing different levels of *Pomc*. (C, D, G, and H) CS revealed shifts in levels of both *Gh* and *Pomc*. (C and G) Average mRNA levels of *Gh* (A) and *Pomc* (C) shown in means +/− SE. (D and H) Percent of CS expressing different levels of *Gh* (D) and *Pomc* (H). Abbreviations: CORT, corticosterone; CS, cortico-somatotroph; *Gh*, growth hormone; *Pomc*, proopiomelanocortin.

The gene expression profiles in somatotrophs were further characterized. A Venn diagram was generated revealing the percent of somatotrophs expressing pituitary-specific transcription factor 1 (*Pou1f1*) and select hypophysiotropic factor receptors, including *Ghrhr*, thyrotropin-releasing hormone receptor (*Trhr*), and somatostatin receptor 2 (*Sstr2*) (Fig. 2). Under basal conditions, ∼50% of the somatotrophs were expressing only *Pouf1f1*, and another ∼40% of the somatotrophs were expressing both *Pouf1f1* and *Ghrhr*. Following CORT treatment, the percent of somatotrophs expressing *Pouf1f1* alone remained unchanged; however, the population of somatotrophs coexpressing *Pouf1f1* and *Ghrhr* under basal conditions decreased by about 50%. There were little to no somatotrophs expressing *Trhr* and *Sstr2* under either condition. The expression of *Gh* was determined, and there was no difference in average mRNA levels between treatments (Fig. 3A). However, when the percent of somatotrophs were separated by expression levels, it revealed a linear decreasing trend (R^2^ = 0.7458) where most of the somatotrophs were expressing low levels of GH (Fig. 3B). CORT treatment increased *Gh* expression levels, but, different from the basal somatotrophs, CORT-treated somatotrophs revealed a polynomial increasing trend (R^2^ = 0.6145) (Fig. 3B). These results suggest a heterogeneity within the somatotroph population, where somatotrophs express vastly different levels of *Gh* mRNA. The heterogeneity can also be visualized through the distribution of the *Gh* gene at different threshold minimums (Fig. S7) (20).

### Lineage Defining Transcription Factors

The expression of transcription factors involved in hormone-producing cell differentiation was investigated. Neuronal differentiation 1 (*Neurod1*) was largely expressed in all the hormone-producing cells, while *Neurod4* was not expressed in any cell type (Fig. S8A) (20). T-box transcription factor 19 (*Tbx19*) was expressed mostly in the corticotrophs with minimal expression localized in the premature nebulous, and *Tbx20* expression was localized more in the thyrotrophs and gonadotrophs (Fig. S8B) (20). Steroidogenic factor 1 (*Nr5a1*) was expressed in the gonadotrophs, and liver receptor homolog-1 (*Nr5a2*) was scarcely expressed in the somatotroph population (Fig. S8C) (20). Forkhead box protein O1 (*Foxo1*) was found in somatotroph, corticotroph, and the premature nebulous clusters (Fig. S8D) (20). *Foxp2* was found in thyrotrophs and gonadotrophs (Fig. S8D) (20). Similar expression levels and distributions were observed in CORT-treated cells (Fig. S8) (20).

### Cortico-somatotrophs Are a Multihormone-producing Cell Type

**Figure 4.**
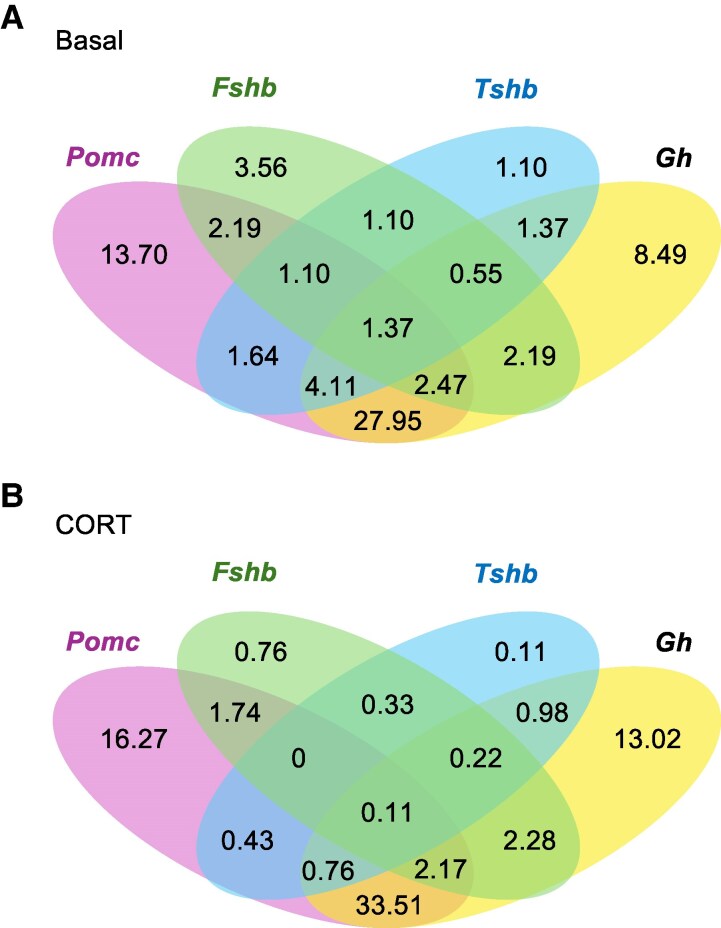
Venn diagram showing percentages of *Pomc*, *Fshb*, *Tshb*, and *Gh* expressing cells in the premature nebulous cluster for basal (A) and corticosterone-treated (B) day 11 chicken embryonic pituitary cells. Cells in the premature nebulous cluster may express more than 1 hormone producing cell marker genes. Abbreviations: *Fshb*, FSH subunit β; *Gh*, growth hormone; *Pomc*, proopiomelanocortin; *Tshb*, TSH subunit β.

The premature nebulous cluster was dissected to determine the percentage of cells expressing *Pomc*, *Gh*, *Fshb*, and *Tshb* (Fig. 4). The population of cells expressing *Fshb* or *Tshb* were low compared to cells expressing *Pomc* or *Gh*. A large population of cells was identified to concurrently express both *Pomc* and *Gh* (∼30%). This cell population was named the cortico-somatotrophs. There was no apparent CORT effect on the percentage of cells expressing the genes for each major endocrine hormone within the premature nebulous cluster.

We further isolated the premature nebulous population and then reclustered these cells and generated UMAPs for visualization (Fig. S9A) (20). Two nondistinct cell clusters were shown under basal conditions, and the cortico-somatotrophs were largely distributed in cluster 1 (Fig. S9B) (20). The distribution of basal cortico-somatotrophs was similarly reflected in both *Gh* and *Pomc* mRNA expressions (Fig. S9C) (20). The DEGs in clusters 1 and 2 were identified (Table S3) (43). With CORT treatment, 3 clusters were revealed, and the clusters were more distinctly separated compared to the clusters seen under basal conditions (Fig. S9A) (20). Out of the 3 clusters, cortico-somatotrophs were distributed across cluster 2 and half of cluster 1 (Fig. S9B) (20). The distribution of CORT-treated cortico-somatotrophs was similarly reflected in *Gh* mRNA expression. Interestingly, *Pomc* mRNA was not only found in the cortico-somatotrophs but also across the rest of cluster 1 (Fig. S9C) (20). The DEGs in clusters 1 and 3 were identified (Table S3) (43). No significant DEGs were identified in cluster 2.

The cortico-somatotrophs were further characterized by comparing this cell population to both the corticotrophs and somatotrophs. Common DEGs were shared between cell types, including *Pomc* and *Pcsk1* compared to corticotrophs and *Ghrhr* and *Enpp2* compared to somatotrophs (Table 3). There were also genes shared among all 3 cell populations, including neuronal vesicle trafficking associated 2 (*Nsg2*) and serpin family I member 1 (*Serpini1*) (Table 3). Using genes for receptors that have been well described to be unique for corticotrophs and somatotrophs, the population of cortico-somatotrophs expressing these genes were compared to the corticotrophs and somatotrophs (Table 4). Under basal conditions, genes for glucocorticoid receptor (*Gr*) and corticotropin-releasing hormone receptor 1 (*Crh-r1*) were expressed in subpopulations of the corticotrophs. In the somatotrophs, *Gr* was also found to be expressed but at a lower percent population, whereas *Ghrhr* was expressed in a higher percent population. Similar to both cell types, *Gr*, *Crh-r1*, and *Ghrhr* were expressed in cortico-somatotrophs, and the percent population of cortico-somatotrophs expressing these genes was at a comparable level to either the corticotrophs or somatotrophs. After CORT treatment, the population of corticotrophs expressing *Gr* and *Crh-1* were significantly reduced compared to basal conditions. Populations of somatotrophs expressing *Gr* and *Ghrhr* were also reduced in response to CORT. Similarly, comparable decreases in cortico-somatotrophs expressing *Gr*, *Crh-r1*, and *Ghrhr* were observed. There was no significant detection of *Crh-r2* and *sstr2* in any of the 3 cell types under either condition. Genes for transcription factors involved in cell-specific differentiation of corticotrophs and somatotrophs were also examined (Table 5). Under basal conditions, a portion of corticotrophs were expressing *Neurod1* (22.12%), *Tbx19* (17.70%), *Foxo1* (23.00%), and *Pit1* (15.04%). A portion of the somatotrophs were also expressing *Neurod1* (31.10%), *Foxo1* (11.84%), and *Pit1* (96.11%). In cortico-somatotrophs, the cell population expressing these genes were comparable to either the corticotrophs or somatotrophs. With CORT treatment, the population of corticotrophs and somatotrophs expressing their corresponding genes were all reduced but at various levels (Table 5). Similar reductions in cortico-somatotrophs expressing these genes were also observed.

**Table 3.** Shared DEGs between CS and corticotrophs or somatotroph cell clusters under basal and CORT-treated embryonic day 11 chicken pituitary cells, relative to all other cell types

Cell clusters	DEGs (basal)	DEGs (CORT)
CS and corticotrophs	*Pomc*, *Pcsk1*, *Calb2*, *Gpx3*, *Rgs2*, *Nsg2*,*^a^ Serpini1*,*^a^ ENSGALG00000035599*, *ENSGALG00000006300*	*Pomc*, *Rasl11a*, *C11orf96*, *Rasd1*, *Nsg2*, *Rgs2*, *Tesc*, *Glul*
CS and somatotrophs	*Ghrhr*, *Enpp2*, *ENSGALG00000000249*, *Rab3c*, *Pkib*, *Olfm1*, *Ank3*, *Scg5*, *Rab3b*, *Nsg2*,*^a^Serpini1*,*^a^ Epas1*, *Scg3*, *Cpe*	*Npy*, *ENSGALG00000033925*, *Tubb3*

Abbreviations: CORT, corticosterone; CS, cortico-somatotroph; DEG, differentially expressed gene.

^*a*^DEGs shared between all 3 cell types.

**Table 4.** Cortico-somatotrophs characterized based on genes for receptors unique to corticotrophs and somatotrophs, individually

	Genes	Corticotrophs		Somatotrophs		Cortico-somatotrophs	
		# Cells	% Pop.	# Cells	% Pop.	# Cells	% Pop.
Basal	*Gr*	47	41.59	68	12.01	50	49.51
	*Crh-r1*	20	17.70	1	0.18	11	10.89
	*Crh-r2*	3	2.65	8	1.41	4	3.96
	*Ghrhr*	4	3.54	271	47.88	72	71.29
	*Sstr2*	0	0.00	5	0.88	1	0.99
CORT	*Gr*	17	3.82	64	2.44	19	6.46
	*Crh-r1*	2	0.45	0	0.00	2	0.68
	*Crh-r2*	2	0.45	30	1.15	5	1.70
	*Ghrhr*	7	1.57	791	30.21	125	42.52
	*Sstr2*	0	0.00	2	0.07	0	0.00

Number of cells and percent population for basal and corticosterone-treated day 11 chicken embryonic pituitary cells revealed similarity between the cortico-somatotrophs compared to the corticotrophs and somatotrophs.

Abbreviations: *Crh-r1*, corticotropin releasing hormone receptor 1; *Crh-r2*, corticotropin releasing hormone receptor 2; *Ghrhr*, growth hormone releasing hormone receptor; *Gr*, glucocorticoid receptor; *Sstr2*, somatostatin receptor 2.

**Table 5.** Cortico-somatotrophs characterized based on genes for transcription factors involved in differentiation of corticotrophs and somatotrophs in embryonic day 11 chicken pituitary cells under basal and CORT-treated conditions

	Genes	Corticotrophs		Somatotrophs		Cortico-somatotrophs	
		# Cells	% Pop.	# Cells	% Pop.	# Cells	% Pop.
Basal	*Neurod1*	25	22.12	176	31.10	40	39.60
	*Tbx19*	20	17.70	2	0.35	13	12.87
	*Neurod4*	0	0.00	0	0.00	0	0.00
	*Foxo1*	26	23.00	67	11.84	40	39.60
	*Pou1f1*	17	15.04	544	96.11	97	96.04
CORT	*Neurod1*	42	9.44	346	13.22	69	23.47
	*Tbx19*	22	4.94	14	0.53	9	3.06
	*Neurod4*	0	0.00	0	0.00	0	0.00
	*Foxo1*	17	3.82	174	6.65	31	10.54
	*Pou1f1*	51	11.460	1899	72.54	250	85.03

Number of cells and percent population revealed similarity between the cortico-somatotrophs compared to the corticotrophs and somatotrophs.

Abbreviations: CORT, corticosterone; *Foxo1*, forkhead box protein O1; *Neurod1*, neurogenic differentiation 1; *Neurod4*, neurogenic differentiation 4; *Pou1f1*, pituitary-specific transcription factor 1; *Tbx19*, T-Box transcription factor 19.

Next, we compared *Gh* and *Pomc* expression in these cell types. In the cortico-somatotrophs, the average expression of both *Gh* and *Pomc* between treatments was not different (Fig. 3C and 3G). No differences in average gene expression levels were observed in somatotrophs for *Gh* (Fig. 3A) and corticotrophs for *Pomc* (Fig. 3E). When the percent of cortico-somatotrophs were separated into bins of different *Gh* mRNA levels, it revealed an overall shift to the right with CORT treatment, indicating an increase in *Gh* expression (Fig. 3D). This increase in *Gh* levels was similarly observed in somatotrophs in response to CORT (Fig. 3B). Conversely, when the cortico-somatotrophs were separated into bins of different *Pomc* mRNA levels, under basal conditions, a large population of the cortico-somatotrophs were expressing high levels of *Pomc*. In response to CORT treatment, there was a shift to the left, indicating a decrease in *Pomc* expression (Fig. 3H). This decrease in *Pomc* expression levels was similarly observed in corticotrophs in response to CORT (Fig. 3F). These results indicate that the similarities observed between cortico-somatotrophs to both the corticotrophs and somatotrophs are more than just gene expression, but they also share similar functional regulation as seen in their response to CORT.

### Verifying the Existence of the Cortico-somatotrophs

**Figure 5.**
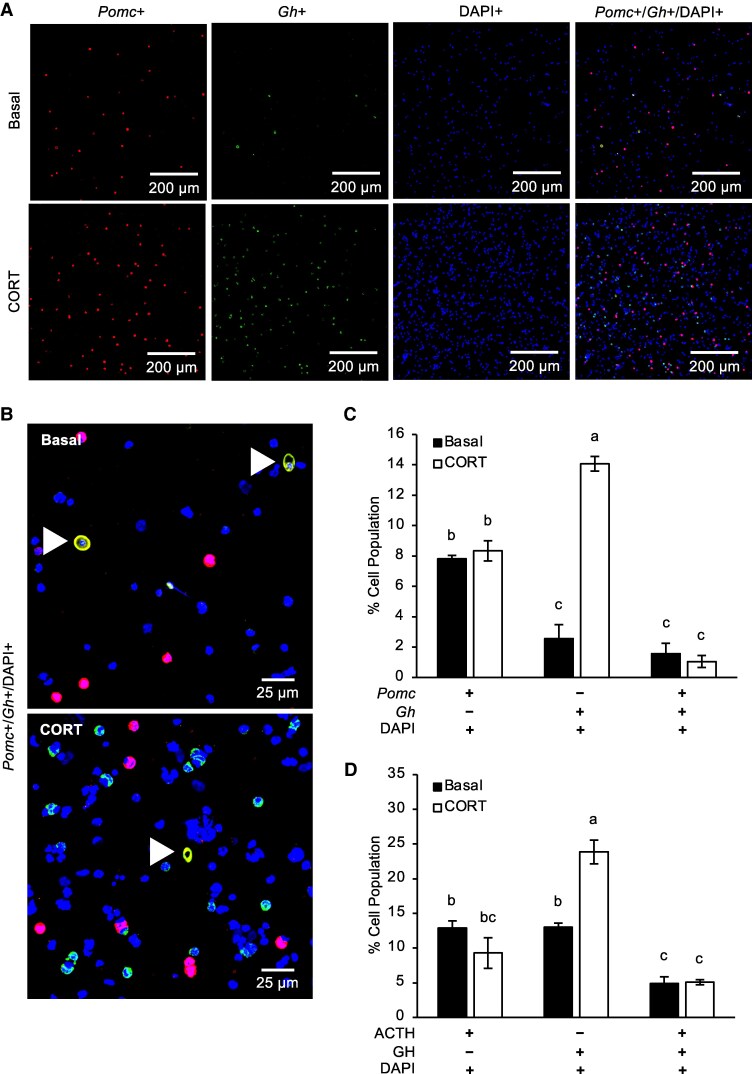
RNA FISH detection of *Pomc* and *Gh* mRNA expression in basal and corticosterone-treated embryonic day 11 chicken pituitary cells. (A) RNA-FISH detection revealed a hormone-producing cell population coexpressing *Pomc* and *Gh* mRNA, named the cortico-somatotrophs. Images were taken at 20× magnification. Scale bars represent 200 μm. n = 4 with 3 technical replicates each but only 1 biological replicate is shown. (B) *Pomc*-producing cells are shown in red. *Gh*-producing cells are shown in green. Cortico-somatotrophs are shown in yellow and indicated with white arrowheads. Images were taken at 20× magnification. Scale bars represent 25 μm. (C) Quantification of RNA-FISH data graphed as percent cell population. Values are LSMeans ± SEM of 4 separate experiments. (D) Quantification of dual-label immunofluorescence detection of ACTH and GH protein expression graphed as percent cell population. Values are LSMeans ± SEM of 4 separate experiments. Significant differences between LSMeans are denoted by different letters (a, b, c). *P < .05. Abbreviations: FISH, fluorescence in situ hybridization; *Gh,* growth hormone; LSMeans, least square means; *Pomc,* proopiomelanocortin.

To confirm the existence of cortico-somatotrophs, e11 CEP cells were visualized at both the mRNA and protein levels using RNA-FISH and dual-label immunofluorescence assays, respectively. Using probes to detect *Pomc* and *Gh* transcripts, corticotrophs (*Pomc*-producing cells) and somatotrophs (*Gh*-producing cells) were visualized in vitro. Along with DAPI staining (detecting the nuclei), a small number of cells were shown to be *Pomc*+, *Gh*+, and DAPI+, indicating the presence of cortico-somatotrophs (Figs. 5A and 5B and S10 and S11) (20). Based on RNA-FISH, the percent population of each cell type was determined (Fig. 5C). Under basal conditions, the corticotrophs (7.83%) were higher than both the somatotrophs (2.55%) and cortico-somatotrophs (1.57%) (*P* < .001). In response to CORT treatment, the corticotrophs (8.34%) and cortico-somatotrophs (1.05%) remain unchanged; however, somatotrophs (14.07%) increased by 5.5-fold (*P* < .0001). Similar results were seen at the protein level. Cortico-somatotrophs were detected in vitro using antibodies for ACTH and GH (Figs. S12-S15) (20). The percent population for each cell type was determined based on dual label immunofluorescence staining (Fig. 5D). There were more corticotrophs (12.86%) and somatotrophs (13%) compared to cortico-somatotrophs (4.89%) under basal conditions (*P* < .01). With CORT treatment, while corticotrophs (9.29%) showed a numerical decrease (*P* = .25), the somatotrophs (23.86%) increased by 2-fold (*P* < .0001). Cortico-somatotrophs remained unchanged (5.08%) in response to CORT. These results verified the existence of cortico-somatotrophs in e11 CEP cells.

## Discussion

We utilized scRNAseq to elucidate the transcriptomes of day 11 chicken embryonic AP cells. During chicken embryonic development, hormone-producing cell types have not yet fully differentiated, and this was seen in nondistinct clusters of cells. Manually assigning cells on a region of the UMAP based on the expression of known cell type markers allowed us to generate cell populations that were expected based on the expression of known genes. We identified 4 endocrine cell clusters, including somatotrophs (*Gh*), corticotrophs (*Pomc*), gonadotrophs (*Fshb*), and thyrotrophs (*Tshb*). These classical endocrine cell types, including lactotrophs (*Prl*), have been identified and somewhat characterized in select vertebrate species (8, 10-16). Somatotrophs are typically only starting to form at this developmental stage; however, we observed a greater abundance of somatotrophs in this study. An explanation for this difference is the increased sensitivity of scRNAseq for detecting mRNA transcripts, which enables earlier and more accurate identification of emerging somatotrophs. Our data did not reveal a clear lactotroph population, because lactotroph differentiation occurs during the late embryonic period (around e18 in chickens) (40, 44). We did, however, detect a small population of somatotrophs that also expressed low levels of *Prl*; we labeled this group of *Gh*- and *Prl*-expressing cells as the mammosomatotrophs (45). Comparing the percent cell population between the endocrine cell types, somatotrophs were the most abundant, which is consistent with the observations seen in rodents (10, 11).

scRNAseq analysis of adult chicken pituitaries showed distinctly separated cluster of cells (14). Despite a lack of well-defined cell clusters, many DEGs were still identified between the endocrine cell types in the present study. With that said, not all identified DEGs were great marker genes, as their expression did not colocalize with known marker genes that we utilized to label each cell type. We identified 1 or more potential gene markers for somatotrophs (*Ghrhr*, *Enpp2*), corticotrophs (*Pcsk1*), and gonadotrophs (*Gal*, *Cart*). There were no other marker genes for thyrotrophs except *Tshb*, and only *Prl* was differentially expressed in mammosomatotrophs.

Glucocorticoids (GCs) can induce premature somatotroph differentiation during development in fetal rat (46), as well as in embryonic mice and chicks (18, 47-51). Since this GC-induced effect is seen in as early as e11 embryos (35, 52, 53), we utilized in vitro CORT treatment on dispersed CEP cells to further characterized each of the endocrine cell types, especially the somatotrophs. Our scRNAseq analysis revealed an increase in somatotroph cell population by ∼9% in response to CORT (Table S2) (43). This was expected as dispersed CEP cells treated with CORT showed an increase in somatotroph abundance, and these cells were further characterized by an increase in GH mRNA, protein, and their capacity to secrete GH (35-37, 39). This suggests a subpopulation of multipotent cells that can be CORT-stimulated to become GH-producing cells. Interestingly, we observed a CORT-induced increase in the gonadotroph population; although inhibitory effects of GCs on gonadotropin secretion have been well documented in various species (54-56).

We examined the CORT effect on gene expression profiles of different pituitary cell types. Visually, the cell clustering patterns were mostly similar between the basal and CORT--reated groups, with the exception of the gonadotroph cluster. There was a CORT-induced shift to where the gonadotroph cluster became nested in between the other endocrine cell clusters. This shift in gonadotroph population on the UMAP suggests changes in its gene expression profile are more similar to other hormone-producing cell types under CORT treatment. Interestingly, there were no DEGs identified between the basal and CORT-treated gonadotrophs. We did, however, identify DEGs between the basal and CORT-treated groups in both the corticotrophs (*Cacna2d1* and *Hnrnpr*) and somatotrophs (*Npy* and *Nsg1*).

In chickens, as in most vertebrate species, the primary stimulators of GH secretion are GHRH and thyrotropin-releasing hormone (57-60, 64), while somatostatin acts an inhibitor of GH release (61, 63). Responsiveness to these stimulatory and inhibitory factors suggest the expression of *Ghrhr*, *Trhr*, and *Sstr2* in the somatotrophs (63). From our scRNAseq analysis, *Ghrhr* was largely expressed by ∼40% of the somatotrophs, which is in line with the ∼50% of embryonic somatotrophs detected to be responsive to GHRH using a reverse hemolytic plaque assay (64). Similarly, *Trhr* was detected in ∼7% of the somatotroph population, and only 15% to 30% of the embryonic somatotrophs responded to thyrotropin-releasing hormone using reverse hemolytic plaque assay (64). These results reveal heterogeneity within the somatotroph population, where not all somatotrophs are responsive to the same stimuli. SSTR2 was shown to be detected in somatotrophs during late embryonic development of chickens (61), but it was undetected (<1%) in e11 CEP cells. In response to CORT, somatotrophs coexpressing *Ghrhr* and *Pou1f1* were reduced by ∼50%. While our study revealed a decrease in *Pou1f1* mRNA, POU1F1 protein and POU1F1-containing cells in e11 CEP were shown previously to be unaffected by CORT treatment (62). This reduction, occurring only at the transcript level, may reflect negative feedback regulation where a reduction in protein abundance is not observed immediately. Notably, CORT had no effect on somatotrophs expression *Pou1f1* alone, suggesting that GC-mediated negative regulation of *Pou1f1* expression may require coexpression of the *Ghrhr* gene. Further analyzing the heterogeneity within the somatotroph population, we separated the somatotrophs by expression levels of *Gh* mRNA, and our analysis revealed a wide range of cells with different levels of *Gh* expression. Under basal conditions, the number of somatotrophs decreased as the level of *Gh* increased in a somewhat linear trend. In response to CORT, the trend was altered, and the number of CORT-induced somatotrophs increased as the level of GH increased. This shift in the distribution of the somatotroph population based on *Gh* mRNA levels indicates that not all somatotrophs are inducible by CORT, which further supports the idea that multiple subpopulations exist within the somatotroph population.

During mammalian pituitary gland development, the first functional hormone-producing cells to differentiate are the corticotrophs that appear at e12.5 of the mouse pituitary (65). The transcription factor TBX19 plays a role specific in driving pituitary progenitor cells toward corticotroph differentiation, but it is not required for commitment (66). In line with TBX19 expression in mice, *Tbx19* was localized in the corticotroph population in e11 CEP cells. Along with *Tbx19* expression, NEUROD1, another cell-specific transcription factor for the *Pomc* gene (67), was also expressed in the corticotrophs. In mice, NEUROD1 deficiency has been shown to cause a delay in *Pomc* expression during early corticotroph differentiation (68). Additionally, NEUROD1 and TBX19 work in transcriptional synergism in stimulating *Pomc* gene expression (67, 69). Interestingly, the expression of *Neurod1* in e11 chicken pituitary cells was not specific to the corticotrophs, but it was expressed in all the hormone-producing cell types. This alludes to the potential role of NEUROD1 in cellular differentiation in other endocrine cells in the chicken pituitary. Through a knockout mouse model, NEUROD4, another member of the NeuroD family, was shown to be essential for normal somatotroph function, although specific targets of this protein are unknown (70, 71). Our scRNAseq data revealed no expression of NEUROD4 in e11 CEP cells. The expression of *Neurod1* in somatotrophs may have similar functional roles as *Neurod4* in mice, which serves to promote somatotroph differentiation in chickens. Furthermore, the shared *Neurod1* expression across all of the hormone-producing cell types suggests that the corticotrophs may not be as distinctly different compared to other cell types in the embryonic chicken. Another T-box transcription factor, TBX20, was shown to be localized in thyrotroph and gonadotroph populations. Similar to *Pomc* regulation by TBX19 and NEUROD1 in the corticotrophs (67, 69), TBX20 may act similarly as TBX19 and work together with NEUROD1 in promoting thyrotroph and gonadotroph differentiation in chickens.

In line with the mice pituitary cell differentiation, *Nr5a1* was expressed and localized in the gonadotrophs. *Nr5a*1 is known to be a transcriptional factor in the development of steroid hormone-producing cells and to be preferentially expressed in gonadotrophs (72, 73). Its family member, *Nr5a2*, was also found to be expressed in the chicken pituitary but more specifically in the somatotroph cluster. The expression of liver receptor homolog 1 (LRH-1) was described as primarily in the liver, pancreas, gastrointestinal tract, and reproductive organs (74). It was later identified in anterior pituitary gonadotrophs (75). LRH-1 was shown to have a direct physical interaction with the glucocorticoid receptor (GR), mediating a reciprocal inhibition of both transcription factors in T cell acute lymphoblastic leukemia cells (76). Our lab has described an involvement of GR in GC-induced chicken *GH* gene activation in the somatotrophs (77), which suggests a potential role for LRH-1 to downregulate GR action in a negative feedback mechanism. The involvement of LRH-1 in the pituitary, specifically in somatotroph differentiation, remains to be characterized.

Another transcription factor that has been identified to be involved in somatotroph differentiation is FOXO1. Nuclear FOXO1 protein was detected in about 40% of the somatotrophs and approximately 10% of gonadotrophs, corticotrophs, and thyrotrophs in embryonic mice pituitary cells at e18.5 (78). In contrast, our scRNAseq data shows *Foxo1* expression mostly localized in the somatotrophs and corticotrophs. Conditional deletion of FOXO1 from the AP in mice resulted in delayed somatotroph differentiation (79). In vivo, loss of FOXO1 impairs the ability of GC to prematurely induce terminal differentiation of mouse somatotrophs (50). Further deletion of both FOXO1 and FOXO3 have reduced expression of important somatotroph genes including *Neurod4*, *Pou1f1*, *Ghrhr*, and GH secretagogue receptor, which led to impaired growth in mice (80). The role of FOXO1 in corticotrophs remains to be described, as this is the first study revealing FOXO1 expression in a large population of the corticotrophs. Another member of the FOX family, FOXP2, was found to be largely expressed in the thyrotrophs and gonadotrophs in e11 CEP cells. FOXP2 was initially characterized as a transcription factor linked to speech and language disorders (81, 82), but recent scRNAseq analyses have revealed that *Foxp2* is also expressed in hormone-producing cells. In line with our findings, *Foxp2* was expressed in both the thyrotrophs and gonadotrophs in adult layers (14). However, in embryonic mice, *Foxp2* was only found to be enriched in the gonadotrophs (11). In adult male zebrafish, FOXP2 deficiency disrupts gonad development and modulates the hypothalamic-pituitary-gonadal axis, especially the regulation of LHβ and FSHβ expression (83). Our finding further supports the newly identified role of FOXP2 in the regulation of reproduction in vertebrates. The expression of *Foxp2* in thyrotrophs appears to be unique to the chicken pituitary, and more research is required to determine its role in thyrotroph functions.

We identified a sixth endocrine cell cluster expressing 2 or more endocrine cell marker genes, namely the premature nebulous. These cells demonstrate a degree of plasticity by producing more than 1 hormone, suggesting the existence of shared progenitor cells that give rise to multiple specialized cell types in the anterior pituitary gland. Identified multihormonal/bipotent anterior pituitary cells include mammosomatotrophs (45), thyrosomatotrophs (84), and corticotrophs that also transiently express either *Prl* (85) or gonadotroph hormones *Lh* and *Fsh* (86). Our scRNAseq data revealed a large population of cells from the premature nebulous cluster that express both *Pomc* and *Gh* concurrently. Dual expression of *Pomc* and *Gh* by pituitary cells has been observed under altered physiological conditions like a pituitary adenoma (87). However, this is the first study suggesting the existence of pituitary cells coexpressing *Pomc* and *Gh* under normal development in vertebrates. We named this pituitary cell type the “cortico-somatotrophs.”

Cell lineages of the 5 classical hormone-producing cell types have been well characterized (88). It is widely accepted that the corticotrophs and the cells derived from the POU1F1 lineage, such as somatotrophs, diverge during early pituitary development (88-90). The transitional intermediate states in these hormone-producing cell lineages were revealed and characterized by a recent scRNAseq analysis (8). However, there is no clear evidence for crossing over between the corticotrophs and somatotrophs during cell differentiation. Further verifying the existence of the cortico-somatotrophs, we visualized a population of cells (∼5%) coexpressing *Pomc*/ACTH and GH at both the mRNA and protein levels through RNA-FISH and dual-label immunofluorescence staining, respectively. Our discovery of the cortico-somatotrophs challenges the current dogma on pituitary cell lineages. Our finding of a large population of functional cortico-somatotrophs during chicken embryonic development indicates that the developmental pathways between corticotrophs and somatotrophs are not as distinctly different as previously proposed (2, 5, 59, 88-90).

We began characterizing the newly identified cortico-somatotrophs by examining their gene expression profiles. In addition to known marker genes, these cells also shared DEGs with both corticotrophs (*Pcsk1*) and somatotrophs (*Ghrhr* and *Enpp2*), suggesting that they possess molecular features of both lineages. Notably, *Pcsk1* and *Ghrhr* play essential roles in regulating corticotroph-specific and somatotroph-specific functions, respectively, underscoring the hybrid identity of this cell type. Consistent with this, cortico-somatotrophs expressed receptor genes characteristic of the 2 lineages: *Crh-r1* for corticotrophs and *Ghrhr* for somatotrophs, supporting their potential capacity to respond to both CRH and GHRH. This dual responsiveness highlights a key novel finding of our study: the discovery and functional characterization of cortico-somatotrophs, a previously unrecognized pituitary cell population that has the potential to integrate corticotroph and somatotroph signaling.

Following CORT treatment, cells expressing *Crh-r1* were barely detected in both corticotrophs and cortico-somatotrophs. Similarly, cells expressing *Ghrhr* in both somatotrophs and cortico-somatotrophs were reduced by ∼30%. These reductions likely reflect negative feedback from downstream CORT stimulation, consistent with known downregulation of *Crh-r1* (91) and *Ghrhr* (92) expression. Additionally, CORT-induced pituitary cells revealed a trend of decreasing *Pomc* gene expression in corticotrophs and cortico-somatotrophs and an increase in *Gh* gene expression in somatotrophs and cortico-somatotrophs. Together, these findings indicate that cortico-somatotrophs are functionally positioned to act as both a corticotroph and a somatotroph simultaneously. This extends the previously known CORT-dependent increase in somatotroph abundance by revealing the existence and dual functionality of this intermediate cell population.

The cortico-somatotrophs were also expressing genes for transcription factors that are critical for cell-specific differentiation for both the corticotrophs and somatotrophs. NEUROD1 and TBX19 are differentially expressed in *Pomc*-positive cells and were identified to promote corticotroph cell differentiation (67, 69), but they were also found to be expressed in the cortico-somatotrophs. Similarly, a significant population of the cortico-somatotrophs were also expressing transcription factors that are essential for normal somatotroph differentiation, including FOXO1 (75, 79) and POU1F1 (93). Interestingly, *Neurod1*, *Foxo1,* and *Pou1f1* were expressed in all 3 cell types. Sharing genes for cell type-specific differentiation suggests the cortico-somatotrophs have the capacity to differentiate into either cell type and that they may be transient precursors to both the corticotrophs and somatotrophs.

Collectively, this study presents a comprehensive view of CEP cells, detailing their composition, heterogeneity, and transcriptomic profiles. The discovery of cortico-somatotrophs suggests a shared lineage between the corticotrophs and somatotrophs, contributing to the concept of pituitary cell plasticity and challenging the current dogma on pituitary cell lineage. Although our conclusions are derived from cultured CEP cells, we acknowledge that in vitro systems may not fully recapitulate the state of these cells in the intact pituitary. Nonetheless, the identification of cortico-somatotrophs in culture provides a strong foundation for future research, especially given the established responsiveness of CEP cells to CORT stimulation and the developmental parallels observed in vivo. In conclusion, by mapping the transcriptional landscape and identifying cortico-somatotrophs as a hybrid/intermediate cell type, we have furthered our understanding of the mechanisms underlying the development and function of the embryonic AP gland.

## Data Availability

Original data generated and analyzed during this study are included in this published article or in the data repositories listed in References.
